# Identification of Active Compounds of *Centipeda minima* for Potentiating Tumor Immunotherapy in Non‐Small Cell Lung Cancer via FANCD2‐cGAS/STING/IFN‐I Signaling Axis

**DOI:** 10.1002/ptr.70460

**Published:** 2026-09-24

**Authors:** Han‐Chen Wang, Chu‐Ying Chen, Jiang‐Tao Xiao, Rou‐Qiao Zheng, Wen‐Da He, Zi‐Wei Xu, Su‐Fen Wei, Tao Su, Guang‐Biao Zhou, Yong‐Qiang Liu

**Affiliations:** ^1^ Key Laboratory of Chinese Medicinal Resource From Lingnan, Ministry of Education, School of Pharmaceutical Sciences Guangzhou University of Chinese Medicine Guangzhou China; ^2^ Research Center of Chinese Herbal Resource Science and Engineering, School of Pharmaceutical Sciences Guangzhou University of Chinese Medicine Guangzhou China; ^3^ Department of Gastroenterology The Second Affiliated Hospital of Guangzhou University of Chinese Medicine (Guangdong Provincial Hospital of Chinese Medicine), Guangdong Provincial Academy of Chinese Medical Sciences Guangzhou China; ^4^ Institute of Cancer Research Henan Academy of Innovations in Medical Sciences Zhengzhou China; ^5^ Key Laboratory of Chronic Disease Prevention and Control of Traditional Chinese Medicine of Guangdong Higher Education Institutes Guangzhou University of Chinese Medicine, KLGHEI (2024KSYS024) Guangzhou China

**Keywords:** 3‐*O*‐methylquercetin, FANCD2, immunotherapy, the ethanol extract of *C. minima*, type‐I interferons

## Abstract

Activation of cGAS/STING/IFN‐I signaling pathway serves as a potential therapeutic strategy to enhance CD8^+^ T cells infiltration and augment antitumor immunotherapy. The medicinal herb *Centipeda minima* has been traditionally employed to treat respiratory diseases and cancer; however, whether *C. minima* and its bioactive constituents can activate CD8^+^ T cell‐mediated antitumor immunity in non‐small cell lung cancer (NSCLC) remains elusive. In this study, the ethanol extract of *C. minima* (ECM) significantly enhanced the cytotoxic activity of human peripheral blood mononuclear cell‐derived activated T cells and murine primary CD8^+^ T cells against NSCLC cells, and promoted CD8^+^ T cell infiltration in the LLC syngeneic tumor model. RNA‐seq profiling indicated that ECM potentially triggers type‐I interferon (IFN‐I)‐mediated innate immunity in NSCLC. Mechanistically, ECM treatment activated the cGAS/STING pathway in NSCLC cells, as evidenced by increased phosphorylation of TBK1 and IRF3, elevated *IFN‐I* gene expression, and enhanced CD8^+^ T cells activity. Notably, ECM markedly downregulated both mitochondrial and nuclear FANCD2 protein levels, which may facilitate cytosolic release of mitochondrial and nuclear DNA, thereby stimulating the cGAS/STING/IFN‐I signaling axis. ECM‐induced FANCD2 downregulation was markedly reversed by the proteasome inhibitor MG‐132. Combination treatment with ECM and anti‐PD‐1 antibody synergistically suppressed tumor growth in the LLC syngeneic model, accompanied by elevated *IFN‐I* expression and pronounced recruitment of activated CD8^+^ T cells. Moreover, 3‐*O*‐methylquercetin and several sesquiterpene lactones abundant in ECM were identified to downregulate FANCD2, activate TBK1/IRF3 pathway, and enhance *IFN‐I* expression in NSCLC cells. Among these active compounds, arnicolide D promoted FANCD2 degradation in a proteasome‐dependent manner, whereas 3‐*O*‐methylquercetin predominantly suppressed FANCD2 gene expression. Collectively, our findings suggest that FANCD2 may serve as a novel therapeutic target for enhancing antitumor innate immunity, and that *C. minima* along with its active compounds can potentiate antitumor immunotherapy by activating the FANCD2‐cGAS/STING/IFN‐I signaling axis.

## Graphical abstract

Schematic diagram illustrating the underlying mechanisms by which ECM and its active components activate the FANCD2‐cGAS/STING/IFN‐I signaling axis, thus enhancing the therapeutic efficacy of anti‐PD‐1 in NSCLC.

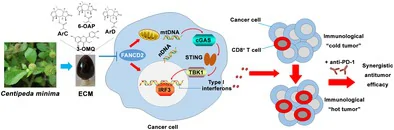

**Abbreviations**
3‐OMQ: 3‐*O*‐methylquercetin
6‐OAP: 6‐*O*‐angeloylplenolin
ALT: alanine transaminase
ArC: arnicolide C
ArD: arnicolide D
AST: aspartate transaminase
cGAS: cyclic GMP‐AMP synthase
CRE: creatinine
ECM: ethanol extract of *Centipeda minima*
GO: Gene Ontology
GSEA: Gene Set Enrichment Analysis
ICA: isochlorogenic acid
ICIs: immune checkpoint inhibitors
IHC: immunohistochemistry
IRF3: interferon regulatory factor 3
NSCLC: non‐small cell lung cancer
STING: stimulator of interferon genes
TBK1: TANK‐binding kinase 1
TME: tumor microenvironment
UA: uric acid
UPLC‐MS: ultra‐performance liquid chromatography‐mass spectrometry

## 1 Introduction

Immune checkpoint inhibitors (ICIs), primarily targeting PD‐1 and PD‐L1, have been shown to significantly improve survival in patients with advanced non‐small cell lung cancer (NSCLC). Nevertheless, the clinical response rate to ICIs remains limited to approximately 20% in NSCLC patients, largely due to insufficient infiltration of cytotoxic CD8^+^ T cells into tumor tissues (Doroshow et al. 2021). Thus, therapeutic approaches capable of enhancing ICIs responsiveness are urgently needed.

The cGAS‐STING pathway, which is activated mainly through sensing cytosolic double‐stranded DNA (dsDNA), plays a critical role in triggering innate immune responses against infections, inflammation, and cancers (Li and Chen 2018). cGAS activation leads to the synthesis of cGAMP, which in turn activates STING and TBK1, ultimately promoting IRF3‐dependent transcription of type‐I interferons (IFN‐I) (Jiang et al. 2020; Zhang, Shang, et al. 2019). As principal IFN‐I subtypes, IFN‐α and IFN‐β subsequently induce the expression of immunostimulatory genes, thereby facilitating immune cell recruitment and orchestrating antitumor immunity (Deng et al. 2014; Duong et al. 2022). Moreover, the cGAS/STING signal pathway contributes to the activation of adaptive immunity, supporting sustained antitumor effects (Chin et al. 2023). Multiple STING agonists have demonstrated favorable tolerability and durable antitumor activity, either as monotherapy or in combination with ICIs (Amouzegar et al. 2021; Chin et al. 2020), highlighting their potential as promising therapeutic strategies in clinical trials. Moreover, co‐administration of recombinant IFN‐α and ICIs has also been shown to enhance the recruitment of CD8^+^ T cells into the tumor tissue (Hu et al. 2022).

The development of STING agonists faces multiple challenges, including low bioavailability and the induction of acute inflammation as well as PD‐L1 upregulation (Gehrcken et al. 2025; Yan et al. 2023). Therefore, there remains a pressing need to develop novel cGAS/STING agonists. Several DNA damage repair proteins have emerged as promising therapeutic targets in antitumor immunotherapy, as they can elevate cytosolic DNA fragments and thereby activate the cGAS/STING signaling pathway (Shen et al. 2019). The induction of mitochondrial DNA damage has also been shown to robustly stimulate the cGAS/STING pathway (Yin et al. 2023). FANCD2, a core component of the Fanconi anemia (FA) pathway, plays essential roles in DNA damage repair. It has been demonstrated not only to maintain nuclear genomic stability but also to protect nascent mitochondrial DNA from degradation, albeit through distinct molecular mechanisms (Emam et al. 2022; Luzwick et al. 2021). While proteins in the FA pathway have gradually been recognized as potential therapeutic targets (Liu et al. 2020), it remain unclear whether targeting FANCD2 can stimulate cGAS‐mediated innate immunity by inducing cytosolic accumulation of nuclear or mitochondrial DNA.

Numerous evidence indicated that traditional Chinese medicine (TCM) exerts antitumor effects largely through immunomodulation (Wang et al. 2020). Moreover, multiple bioactive compounds derived from TCM have been shown to activate cGAS or STING, thereby enhancing the therapeutic efficacy of ICIs in lung cancer (Gao et al. 2025). *Centipeda minima* (L.) A. Braun & Asch has been widely used in TCM for the treatment of rhinitis, asthma, and other inflammatory respiratory conditions, indicating its potential role in improving the immunological function (Liu et al. 2023; Tan et al. 2022). Notably, *C. minima* also has been documented to treat nasopharyngeal carcinoma (Huang et al. 2013). Our previous studies revealed that the ethanol extract of *C. minima* (ECM) and the active compounds exhibit potential chemosensitizing activity by suppressing the FA pathway (Chen et al. 2024; Fan et al. 2021; Wang et al. 2025). Notably, the aqueous extract of *C. minima* has also been reported to potentiate the efficacy of ICIs in NSCLC treatment; however, whether *C. minima* or its active constituents can activate innate immunity and promote CD8^+^ T cells infiltration into the tumor microenvironment (TME) remains unclear (Wang et al. 2024). In this study, we demonstrated that ECM significantly downregulates both mitochondrial and nuclear FANCD2 protein levels, leading to the cytosolic release of DNA fragments and subsequent activation of the cGAS/STING/IFN‐I signaling axis. This cascade promotes the infiltration of CD8^+^ T cells into the TME. Furthermore, combination therapy with ECM and anti‐PD‐1 antibody exhibited synergistic anti‐NSCLC activity.

## 2 Materials and Methods

### 2.1 Antibodies and Reagents

MSA‐2 (STING agonist), RU.521 (cGAS inhibitor), C‐176 (STING inhibitor) were purchased from Bidepharm (Shanghai, China); carboxyfluorescein succinimidyl ester (CFSE) was sourced from Meilunbio (Dalian, China); Deoxyribonuclease I (DNase I), chloroquine, and MG‐132 were obtained from Solarbio (Beijing, China); Collagenase I, Collagenase II, and Collagenase IV were purchased from Biosharp (Hefei, China); Recombinant Mouse and human IL‐2 was sourced from Novoprotein (Suzhou, China). Anti‐mouse CD3 and anti‐mouse CD28 antibodies obtained from Biolegend (California, USA) were used for primary CD8^+^ T cell activation. Antibodies used for Western blotting include anti‐phospho‐TBK1 (Ser172), anti‐IRF3, anti‐STING, anti‐CD8α, and anti‐IFNγ were obtained from Abclonal (Wuhan, China), anti‐phospho‐IRF3 (Ser396), anti‐TBK1, anti‐VDAC1, and anti‐lamin B1 were obtained from Proteintech (Wuhan, China), and anti‐FANCD2 was from Santa Cruz Biotechnology. Antibodies used for flow cytometry analysis includes anti‐CD16/32, Violet 450 anti‐CD45, APC anti‐CD8α, PerCP/Cyanine5.5 anti‐CD3, PE anti‐Gr‐1, APC anti‐CD11b, FITC anti‐CD69, and PE anti‐IFNγ were obtained from Biolegend (San Diego, USA), these antibodies mentioned above were intended for anti‐mouse applications. Rat anti‐mouse PD‐1 and rat IgG2a isotype control for in vivo treatment were from Leinco (Missouri, USA). Erythrocyte lysis buffer, Mitochondria Isolation Kit, and Nuclear Protein Extraction Kit were obtained from Beyotime (Shanghai, China).

### 2.2 Cell Culture

A549 and H1975 cell lines were from the Cell Bank of the Chinese Academy of Sciences (Shanghai, China). The mouse Lewis lung carcinoma (LLC) cell line was sourced from Jennio Biotech (Guangzhou, China). The H1975 cell line was cultured in RPMI‐1640 complete medium, while LLC and A549 cells were maintained in DMEM complete medium added with 10% fetal bovine serum (Baidi Biotech, Hangzhou, China).

### 2.3 Activation of Human Peripheral Blood Mononuclear Cells and Mouse Primary CD8 ^+^ T Cells

Human peripheral blood mononuclear cells (PBMCs) that obtained from STEMCELL Technologies (Vancouver, Canada) were first stimulated with human CD3/CD28 T Cell Activation Magnetic Beads (MCE, USA), followed by the stimulation of the recombinant human IL‐2 in 1640 complete medium to generate activated human T cells (Zhou et al. 2024), which were used to co‐culture with A549 and H1975 cells.

Mouse CD8^+^ T Cell Separation Kit (RWD Life Science, Shenzhen, China) was utilized to purify the primary CD8^+^ T cells from the spleen of C57BL/6J mice. In brief, after the mouse spleen grinding, erythrocyte lysis was performed, then 1 × 10^7^ cells/100 μL were incubated with biotin‐labeled anti‐Mouse CD8 antibody for 15 min at 4°C and subsequently with Streptavidin magnetic beads to isolate CD8^+^ T cells in a magnetic field. T cells cultured in 1640 complete medium were stimulated with CD3/CD28 antibodies (2 μg/mL) to prime naïve CD8^+^ T cells (Esensten et al. 2016), and then recombinant IL‐2 (10 ng/mL) was used to stimulate the proliferation of T cells.

### 2.4 Co‐Culture of NSCLC Cells and T Cells

H1975 and A549 cells were co‐cultured with human PBMCs with activated T cells, while LLC cell was co‐cultured with activated primary mouse CD8^+^ T cells, respectively, after these primary T cells were pre‐stimulated with CD3/CD28 antibodies and IL‐2. NSCLC cells were pretreated with ECM for 24 h, then the ratio of PBMCs or mouse CD8^+^ T cells to NSCLC cells was set as 15:1 or 5:1, respectively. After the co‐culture for 24 h, T cells and dead cells were carefully removed, the NSCLC cells alive in the plates were incubated with crystal violet (0.05%). Then the cell staining was dissolved with 33% glacial acetic acid, and the percentage of living NSCLC cells was calculated according to the ratio of the absorbance at 570 nm of the treatment group to the control group.

For the detection of the proliferation ratio of CD8^+^ T cells, these cells were pre‐labeled with CFSE (2.5 μM) for 10 min and then cultured in the conditioned medium harvested from ECM‐treated LLC cells for 24 h. CFSE staining intensity was analyzed by using flow cytometry.

### 2.5 Small Interfering RNA Transfection

Small interfering RNA (siRNA) was synthesized by Hongxun Biotech (Suzhou, China). LLC cells were transfected with siRNA (70 pmol/well) in a six‐well plate utilizing Lipomaster 3000 (Vazyme, Nanjing, China). The cells were treated with indicated drugs after the transfection for 2 days. The siRNA sequence for mouse *STING* and human *FANCD2* have been listed in Table S1.

### 2.6 Cell Viability Assay

NSCLC cells (4–5 × 10^3^/well), PBMCs (8 × 10^4^ cells/well), and primary CD8^+^ T cells (8 × 10^4^ cells/well) were cultured in a 96‐well plate and treated with indicated drugs for 48 h, then CCK‐8 was added to measure the absorbance at 450 nm. Relative cell viability was calculated by using the ratio of the absorbance value of the treatment group to the control group.

### 2.7 ECM Preparation and Ultra‐Performance Liquid Chromatography‐Mass Spectrometry Analysis

The herb of *C. minima* was collected from Hubei Province in China and identified by Tongrentang Chinese Medicine Pharmacy, a voucher specimen has been deposited in the Research Center of Chinese Herbal Resource Science and Engineering at Guangzhou University of Chinese Medicine. ECM was prepared according to previous study (Wang et al. 2019); ultra‐performance liquid chromatography‐mass spectrometry (UPLC‐MS) analysis was carried out using the procedure we previously described (Wang et al. 2025). The UPLC mobile phase (0.1% acetic acid [A] and acetonitrile [B]) gradient was: 15% B (0–2 min), 15%–70% B (2–23 min), 70% B (23–28 min), 70%–95% B (28–32 min), 95% B (32–36 min), 95%–15% B (36–37 min). The main compounds in ECM were defined based on the comparison of the retention time of indicated ion chromatograms and molecular mass of these standard reference compounds, which include rutin, caffeic acid, chlorogenic acid, isochlorogenic acid B (ICA‐B), isochlorogenic acid A (ICA‐A), isochlorogenic acid C (ICA‐C), 6‐*O*‐angeloylplenolin (6‐OAP), arnicolide C (ArC) and arnicolide D (ArD), and 3‐*O*‐methylquercetin (3‐OMQ). 3‐OMQ was obtained from Nanjing Jingzhu Biotech (Nanjing, China) and the other compounds were obtained from Weikeqi Biotech (Sichuan, China).

### 2.8 Western Blotting

Protein samples were prepared using RIPA lysis buffer. The denatured proteins were separated using SDS‐PAGE gel electrophoresis and transferred onto a PVDF membrane (Merck Millipore, Darmstadt, Germany), which was blocked with skimmed milk, incubated with specific primary antibodies, and probed with horseradish peroxidase‐conjugated secondary antibodies. Relative protein levels were detected using Biolight Biotech Chemiluminescent System (Guangzhou, China). ImageJ software was used to analyze the protein bands optical density. All reagents above were obtained from FudeBio (Hangzhou, China).

### 2.9 Quantitative Real‐Time PCR and Cytosolic dsDNA Quantitation

Total cellular RNA was extracted by using a Tissue/Cell Total RNA Isolation Kit (Vazyme). ToloScript All‐in‐one RT EasyMix Kit (Tolobio, Shanghai, China) was employed to obtain the cDNA for Quantitative Real‐Time PCR (qRT‐PCR) analysis using the SYBR qRT‐PCR Master Mix Kit (Tolobio, Shanghai, China); *β‐actin* was utilized as the reference gene. Primer sequences have been listed in Table S1.

Cytosolic dsDNA quantitation was performed according to previous study (Lai et al. 2025). After ECM treatment for 24 h, the cells were permeabilized in a NP‐40/digitonin buffer for 30 min. Both mitochondrial and nuclear DNA (mtDNA and nDNA) released into the cytosol can be purified in the supernatant after centrifugation while the total nDNA was extracted from the precipitation, the mitochondrial and nuclear biomarkers, VDAC1 and lamin B1, were examined by western blotting to avoid mitochondria and nucleus contamination in the cytosolic fraction. The cytosolic dsDNA and total nDNA were purified by using a Blood/Cell/Tissue DNA Isolation Kit (TIANGEN, China). Primer sequences that used in this study have been listed in Table S1.

### 2.10 CD8 ^+^ T Cell Migration Assay

LLC‐induced CD8^+^ T cell migration assay was carried out in a Transwell (5 μm pore size, Corning) co‐culture system. LLC cells (1.5 × 10^5^) were plated in a 24 well plate and pretreated with ECM for 24 h. Then, the mouse spleen CD8^+^ T cells (5 × 10^5^) were plated in the upper chamber for another 24 h. T cells that infiltrated into the wells were collected, flow cytometry was performed using CD8^+^ T antibody to quantify the total migration cells.

### 2.11 RNA Sequencing

Tumor tissues of LLC syngeneic model were used to extract total RNA by using Trizol reagent. The RNA samples were qualified and quantified, followed by purification, fragmented into small pieces, cDNA synthesis, and PCR amplification. The final RNA‐seq library sequencing was conducted by using MGIseq2000 platform (BGI‐Shenzhen, China). RNA‐seq data analysis including clustering heatmap generation, Gene Set Enrichment Analysis (GSEA), and Gene Ontology (GO) enrichment plotting were carried out using Dr. Tom Multi‐Omics Data mining system.

### 2.12 Animal Experiments

Animal experiments were carried out in accordance with the procedures and guidelines approved by the Animal Ethical Committee of Guangzhou University of Chinese Medicine (registration NO.: ZYD‐2024‐142). Male C57BL/6 mice (5 weeks old) were from Risemice Biotechnology (Zhaoqing, China) and maintained under specific pathogen‐free conditions. LLC syngeneic model was established by subcutaneous injection of the cells (1.5 × 10^6^ cells per mouse). Once tumor volumes reached approximately 80–100 mm^3^, these mice were randomly assigned to the experimental groups as indicated below. The dosage and vehicle for ECM treatment was selected based our previous study (Wang et al. 2025): vehicle control (10% dimethyl sulfoxide and 90% corn oil), ECM 400 and 800 mg/kg (i.g., daily). For the combination treatment experiments: isotype control (200 μg, i.p., once every 3 days), ECM (400 mg/kg, i.g., daily), anti‐PD‐1 (200 μg, ip, once every 3 days), and the combination with ECM and anti‐PD‐1. The mice were treated with the above drugs for 12 days, body weight and tumor volume were recorded every 2 days, Tumor volume (mm^3^) = (length × width^2^)/2. The serum was harvested to assess uric acid (UA), creatinine (CRE), aspartate aminotransferase (AST), and alanine aminotransferase (ALT) using the kits from Nanjing Jiancheng Bioengineering Institute (Nanjing, China).

### 2.13 Immune Profiling by Flow Cytometry

At the end of the animal experiments, the tumors were cut into pieces and digested using a mixture of collagenase I (0.5 mg/mL), collagenase II (0.1 mg/mL), collagenase IV (0.5 mg/mL), and DNase I (0.1 mg/mL) in L‐15 medium at 37°C for 30 min. After erythrocyte lysis, single‐cell suspensions (1 × 10^6^/100 μL) were first blocked with anti‐CD16/32 for 10 min to prevent antibodies non‐specific binding, followed by the incubation with surface‐labeled antibodies against CD45, CD3, CD8, CD69, CD11b, Gr‐1, and IFNγ for 30 min at 4°C. For the intracellular factor IFNγ staining, the cells were firstly permeabilized by using the Intracellular Fixation/Permeabilization Kit (Elabscience). Flow cytometry was performed and analyzed using a CytoFLEX‐S flow cytometer along with CytExpert software (Beckman Coulter, California, USA). The full flow cytometry gating strategies, including singlet discrimination (SSC‐A vs. SSC‐H), dead cells exclusion using propidium iodide staining, CD45 gating for total leukocyte, CD3 gating for total T cells, and CD11b gating for myeloid‐derived cells, were shown in Figure S1C.

### 2.14 Immunohistochemistry and Immunofluorescence

Immunohistochemistry (IHC) analysis was performed according to standard protocols (Wang et al. 2025). The tissue sections were incubated with primary antibodies against Ki67, CD8, and IFNγ overnight at 4°C, then DAB staining (Solarbio, Beijing, China) and nuclei counterstaining with hematoxylin were conducted, the sections were mounted with neutral resin. Images were then photographed by using Olympus Microscope (BX53, Tokyo, Japan) at a magnification of 40×. These staining‐positive cells were subsequently quantified using ImageJ software.

Immunofluorescence assay using anti‐FANCD2 antibody was performed according to previous study (Chen et al. 2024). After ECM (16 μg/mL) treatment for 24 h, A549 cells were fixed with 4% paraformaldehyde, permeabilized with Triton X‐100 (0.5%) in PBS, and blocked using normal goat serum. Then, the cells were stained with anti‐FANCD2 antibody at 4°C overnight and then with Fluor488 goat anti‐mouse IgG for 2 h. After counterstaining with DAPI, immunofluorescence images were photographed using a confocal microscope (Nikon, Japan), the mean fluorescence intensity of FANCD2 staining was calculated using ImageJ software and normalized to DAPI staining.

### 2.15 Statistical Analysis

All in vitro experiments in this study were replicated a minimum of three times. Data are reported as the means ± standard deviation (SD) for in vitro experiments; while data are shown as the means ± standard error of the mean (SEM) for in vivo experiments. Statistical analyses were carried out using GraphPad Prism 9.0. An unpaired, two‐tailed Student's *t*‐test was used to assess statistical differences between two groups, while one‐way or two‐way ANOVA with subsequent post hoc tests were conducted for multiple groups' comparison. *p* < 0.05 was considered to be statistically significant.

## 3 Results

### 3.1 ECM Activates the Innate Antitumor Immunity In Vivo

We first evaluated the immunomodulatory effects of ECM using LLC syngeneic mouse model. ECM administration significantly suppressed tumor growth (Figures 1A and S1A), without affecting mouse body weight (Figure S1B). Notably, ECM treatment markedly enhanced the tumor infiltration of CD8^+^ T cells (Figure 1B), which exhibited elevated expression of the activation marker CD69 (Figure 1C) (Labiano et al. 2017). In contrast, the proportions of CD8^+^ T cells and CD69^+^CD8^+^ T cells in the spleen showed no significant differences between the ECM‐treated and control groups; whereas ECM treatment substantially reduced the percentage of myeloid‐derived suppressor cells (MDSCs) within tumors, while leaving the frequency of regulatory T (Treg) cells unchanged (Figure S1C–F). To further elucidate the mechanism underlying ECM‐mediated immunomodulation, we performed RNA sequencing (RNA‐seq) analysis of tumor tissues. Principal‐component analysis (PCA) indicated clear separation between control and ECM groups (Figure S1G). GO analysis showed significant enrichment of genes associated with “immune system process,” “cellular response to IFN‐β,” and “innate immune response” following ECM treatment (Figure 1D). Consistently, GSEA revealed strong enrichment of innate immune response‐related gene sets in the ECM group compared to controls (Figure 1E). Heatmap analysis further demonstrated upregulation of genes involved in IFN‐I signaling and T cell activation (Figure 1F). Two key candidate genes, *CD8* and *CD69*, were validated to be significantly elevated in ECM‐treated tumors (Figure 1G). These results demonstrate that ECM treatment remodels the TME by activating IFN‐I‐associated innate immunity.

**FIGURE 1:**
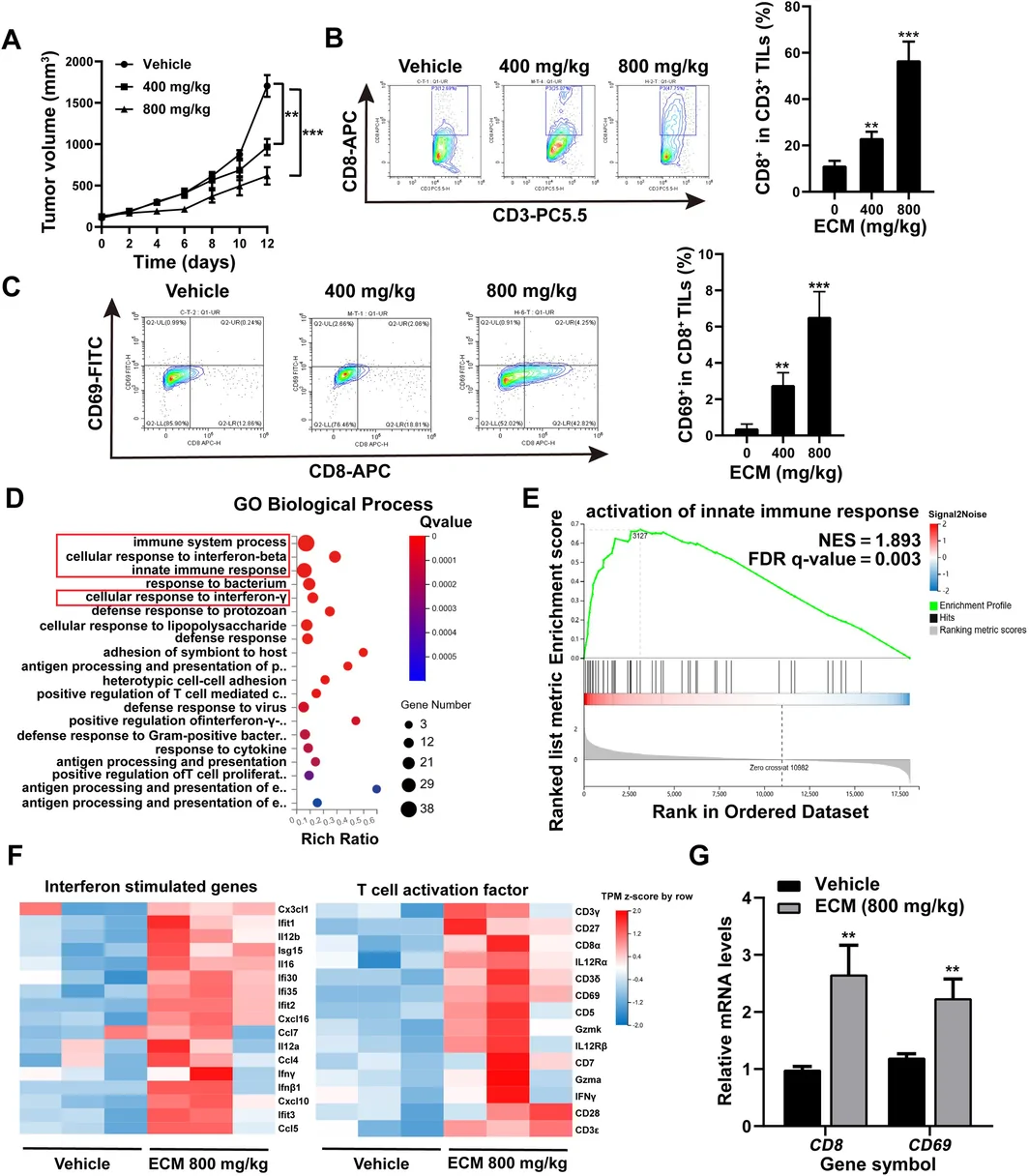
ECM inhibits tumor growth by activating innate antitumor immunity. (A–C) At the end of the animal experiments, tumor volume was calculated (A); flow cytometry analysis was performed to show the percentage of CD3^+^CD8^+^ (B) and CD3^+^CD8^+^CD69^+^ (C) cells among all CD45^+^ tumor infiltrating lymphocytes (TILs) (*n* = 5). (D–F) RNA‐seq was carried out by using tumor tissues from vehicle control and ECM group; GO analysis showed the involved biological process of these differentially expressed genes (D); GSEA showed these differentially expressed genes were highly enriched in innate immune response (E); heatmap analysis was performed to show gene expression differences of T cell activation markers and interferon‐related factors between vehicle control and ECM group (F). (G) qRT‐PCR was performed to examine the expression levels of *CD8* and *CD69* in tumor tissues (*n* = 5). Data presented above are shown as means ± SEM, **p < 0.01, ***p < 0.001 versus control.

### 3.2 ECM Enhances T Cell‐Mediated Cytotoxicity Against NSCLC Cells

Then we evaluated whether ECM enhances T‐cell‐mediated cytotoxicity against NSCLC cells. Based on cell viability assay of ECM (Figure 2A,B), we selected concentrations that showed no significant cytotoxicity toward NSCLC, human PBMCs, or mouse CD8^+^ T cells for subsequent experiments. Co‐culture experiments demonstrated that ECM treatment significantly increased the cytotoxicity of human PBMCs against A549 and H1975 cells (Figures 2C,D and S2A). Furthermore, the C57BL/6 mouse spleen was used to purify naïve CD8^+^ T cells (Figure S2B), which were stimulated with CD3/CD28 antibodies to assess their cytotoxicity against LLC cells. ECM treatment markedly enhanced CD8^+^ T cells‐mediated killing of LLC cells (Figure 2E,F). To further evaluate T cell activation, CD8^+^ T cells were collected from the co‐culture system and analyzed for IFNγ expression by flow cytometry. The results showed that ECM significantly increased IFNγ levels in primary mouse CD8^+^ T cells (Figure 2G). Taken together, these finding indicate that ECM enhances CD8^+^ T cell‐mediated cytotoxicity against NSCLC cells.

**FIGURE 2:**
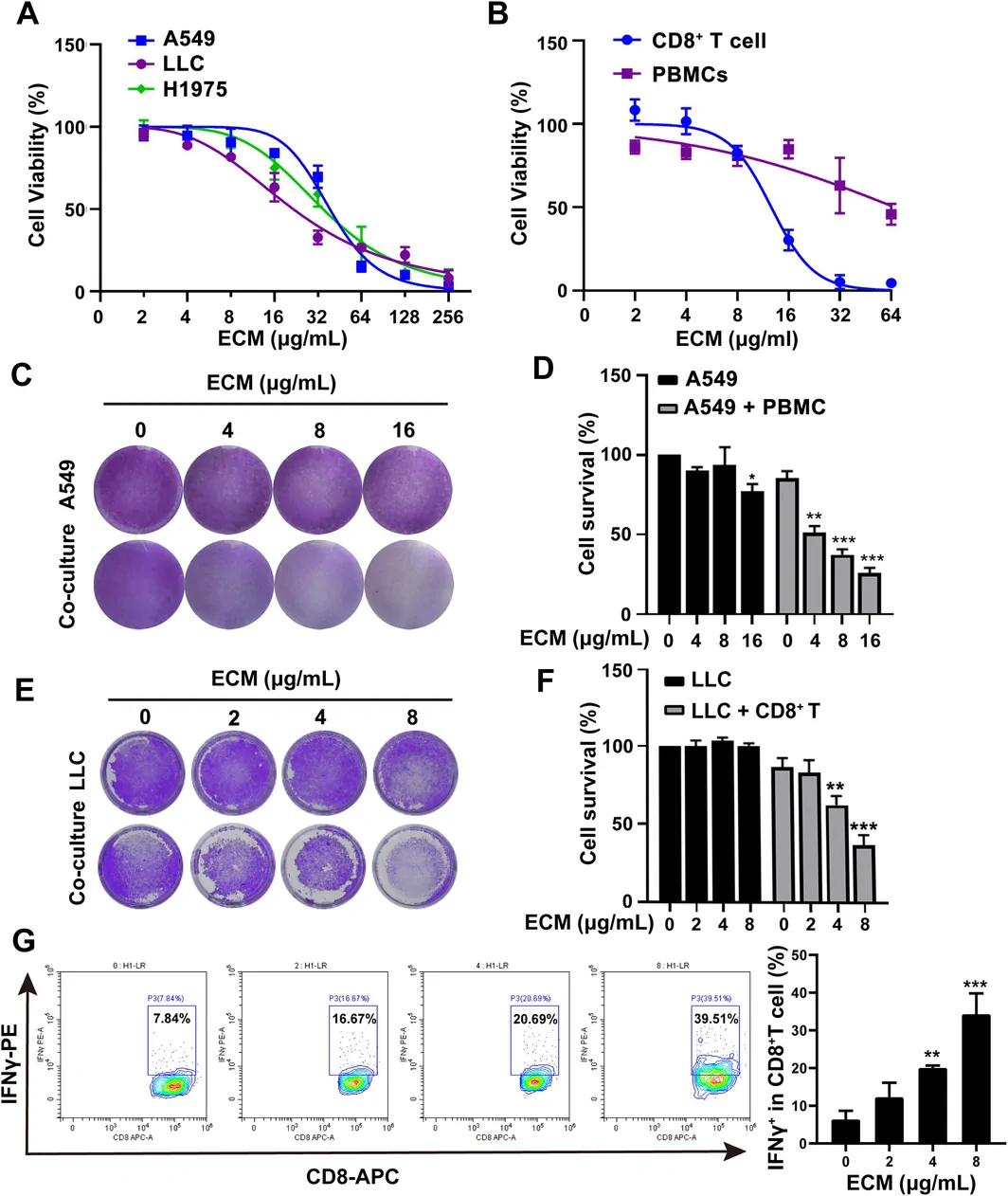
ECM enhances cytotoxicity of T cells against NSCLC cells. (A, B) After ECM treatment for 48 h, CCK‐8 assay was conducted to examine the viability of NSCLC cells (A), mouse CD8^+^ T cells (B), and human PBMCs (B). (C–F) After ECM treatment of NSCLC cells for 24 h, co‐cultured assay was performed for A549 and human PBMCs (C) or LLC and mouse CD8^+^ T cells (E) for an additional 24 h, the viable cancer cells were visualized by crystal violet staining and the percentage of viable cells were calculated according to the absorbance of dissolved crystal violet. (G) Flow cytometry was performed to measure the percentage of IFNγ^+^ among all CD8^+^ T cells. Data shown are means ± SD; *n* = 3, *p < 0.05, **p < 0.01, ***p < 0.001 versus control.

### 3.3 ECM Potentiates IFN‐I Expression via the TBK1/IRF3 Signaling Pathway

To determine whether ECM enhances cGAS/STING pathway‐dependent IFN‐I expression, we examined the activation of key downstream effectors of STING signaling. ECM treatment robustly stimulated phosphorylation of TBK1 and IRF3 in both A549 and LLC cells (Figure 3A,B). In contrast, ECM did not activate NF‐κB (Figure 3C) or upregulate PD‐L1 expression (Figure S3A) in NSCLC cells, ECM also showed no apparent effect on upregulating PD‐L1 in tumor tissue from LLC syngeneic model (Figure S3B). As expected, ECM dose‐dependently increased the mRNA levels of both *IFNα2* and *IFNβ1* genes in NSCLC cells (Figure 3D,E), without significantly altering expression of the NF‐κB‐responsive gene *IL‐6* (Figure S3C). These results indicate that ECM specifically activates TBK1/IRF3 rather than the TBK1/NF‐κB pathway, downstream of STING. We further investigated whether ECM‐treated tumor cells enhance the activity of primary CD8^+^ T cell. In transwell migration assay, ECM‐treated LLC cells significantly promoted CD8^+^ T cells migrate across the well of the insert (Figure 3F,G). Moreover, CFSE‐based proliferation assay revealed that ECM markedly increased the proliferation rate of CD8^+^ T cell in co‐culture with LLC cells (Figure 3H). Therefore, these findings suggest that ECM promotes CD8^+^ T‐cell activation by inducing IFN‐I production in NSCLC cells through selective activation of the TBK1/IRF3 signaling axis.

**FIGURE 3:**
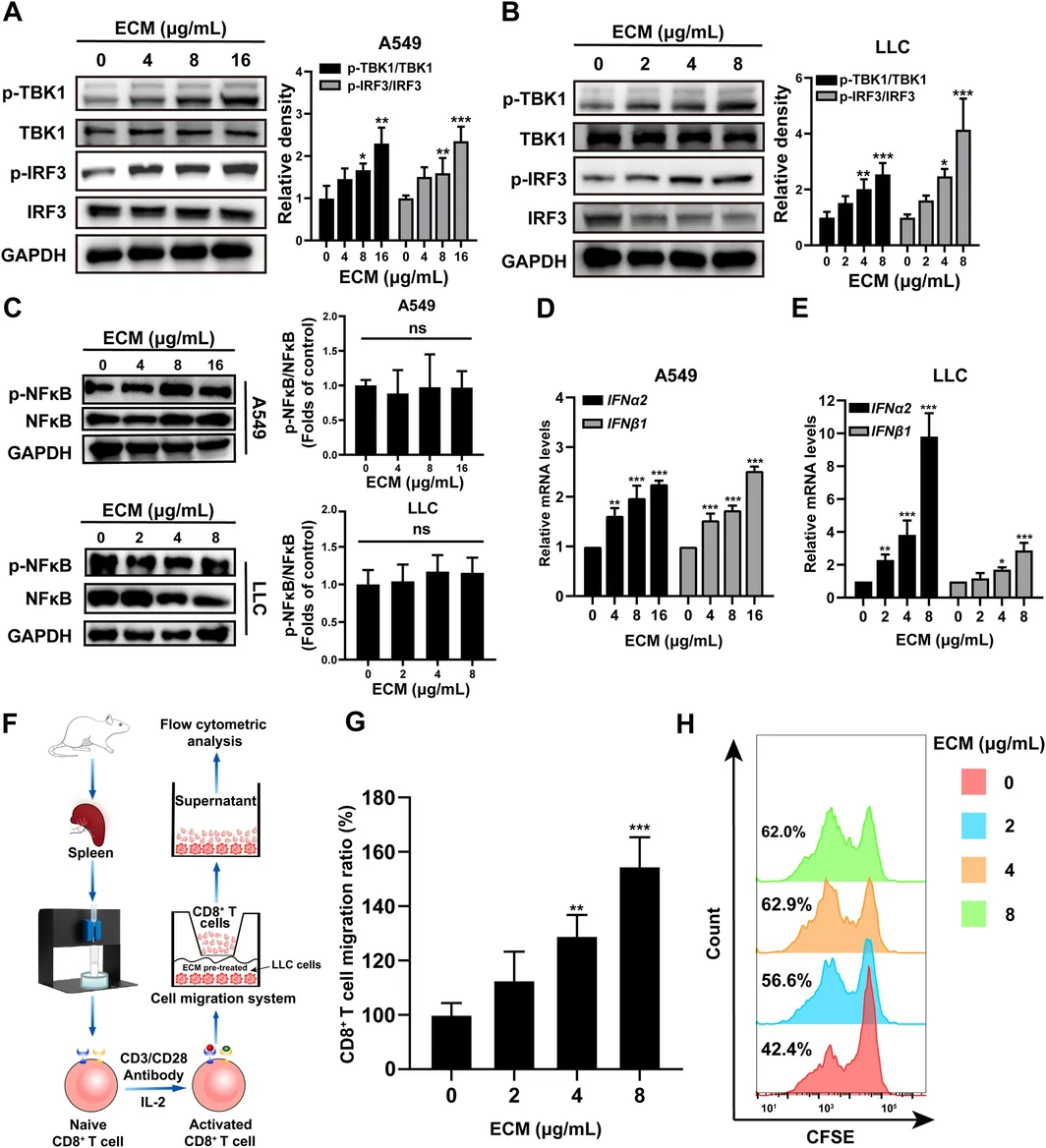
ECM induces the activation of TBK1/IRF3/IFN‐I signaling axis in NSCLC. (A–C) Protein levels of phosphorylated TBK1 (S172), IRF3 (S396), and NFκB (S536) in A549 and LLC cells were examined after ECM treatment for 24 h. (D, E) qRT‐PCR was performed to quantify mRNA levels of *IFNα2* and *IFNβ1* in NSCLC cells after ECM treatment for 24 h. (F) Schematic diagram of primary CD8^+^T cell isolation for the in vitro cell migration assay co‐cultured with LLC cells. (G) CD8^+^ T cells were co‐cultured with LLC cells that were pretreated with ECM for 24 h in the transwell system; flow cytometry was performed to analyze relative migration of CD8^+^ T cells across the transwell. (H) After CFSE labeling, flow cytometry was performed to analyze CD8^+^ T cell proliferation when co‐cultured with LLC cells pretreated with ECM for 24 h. Data shown are means ± SD; *n* = 3; ns, not significantly different; *p < 0.05, **p < 0.01, ***p < 0.001 versus the control group.

### 3.4 ECM‐Induced CD8 ^+^ T Cell Activation Depends on the cGAS/STING Pathway

Given that cytosolic DNA can trigger cGAS activation, we next investigated whether ECM‐stimulated CD8^+^ T cell activation is dependent on the cGAS/STING axis. Notably, ECM treatment induced substantial DNA leakage from both the nucleus and mitochondria; meanwhile, the cytosolic fractions obtained were devoid of nuclear and mitochondrial contamination (Figure 4A), indicating that ECM‐induced genomic and mitochondrial DNA instability may contribute to cGAS activation. As expected, pharmacological inhibition of cGAS significantly attenuated ECM‐induced IRF3 activation (Figure 4B). Correspondingly, siRNA‐mediated knock down of STING (Figure 4C) abolished ECM‐stimulated IRF3 phosphorylation in LLC cells (Figure 4D,E). Furthermore, *STING* knock down in LLC cells substantially impaired the ability of ECM to enhance CD8^+^ T cell‐mediated cytotoxicity in cell co‐culture system (Figure 4F), confirming that ECM‐induced activation of the cytosolic DNA‐cGAS/STING pathway in NSCLC cells is essential for potentiating CD8^+^ T cell activity.

**FIGURE 4:**
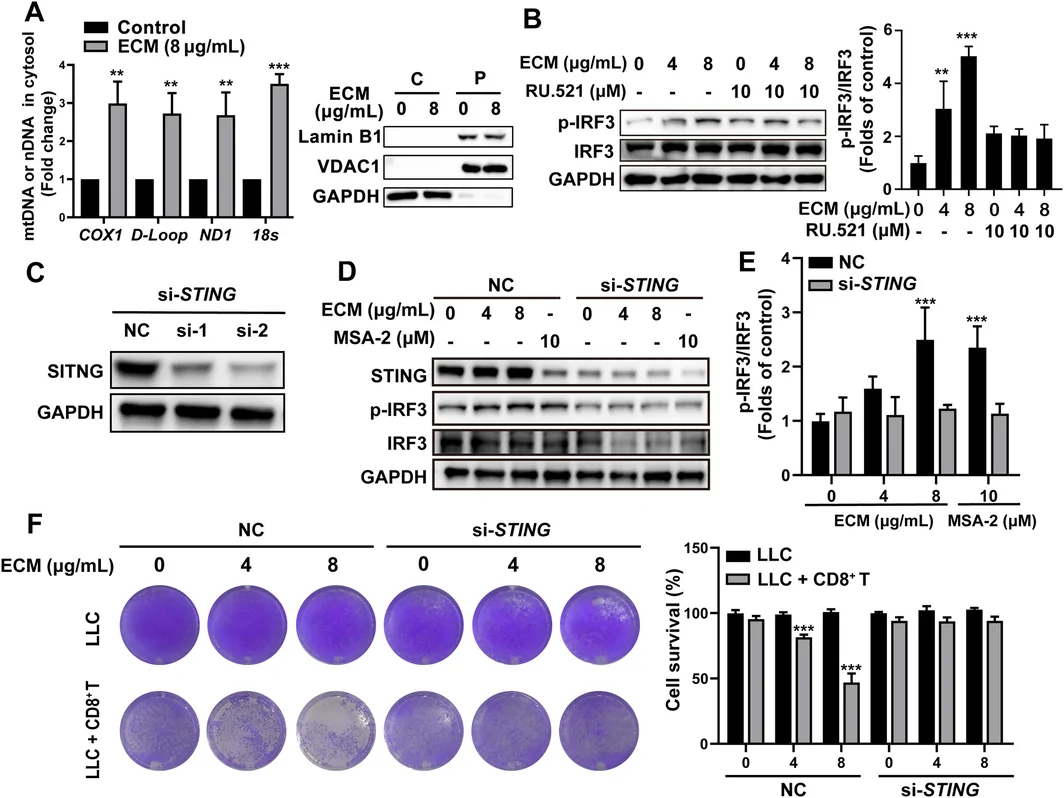
cGAS/STING pathway suppression attenuates ECM‐enhanced cytotoxicity of CD8^+^ T cells. (A) After ECM treatment for 24 h in LLC cells, qPCR was performed (left panel) to examine the relative levels of cytosolic mtDNA (*COX1*, *D‐loop*, and *ND1*) and nDNA (*18S*); the distribution of VDAC1 (mitochondrial marker) and lamin B1 (nuclear marker) was examined in the cytosolic (C) and precipitation (P) fractions by western blotting (right panel). (B) After the combination treatment of ECM with RU.521 for 24 h, Western blotting was performed to examine protein levels of p‐IRF3 and IRF3 in LLC cells. (C) Western blotting was performed to show the knockdown efficacy of si‐*STING* in LLC cells after siRNA transfection for 48 h. (D, E) After ECM or MSA‐2 treatment for 24 h in LLC cells, the protein levels of STING, p‐IRF3, and IRF3 in NC and si‐*STING* group were examined by Western blotting. (F) LLC cells were pretreated with ECM for 24 h and co‐cultured with CD8^+^ T cells for an additional 24 h, crystal violet staining was performed to show viable cells of LLC in NC and si‐*STING* group; the percentage of survival cells were calculated according to absorbance of dissolved crystal violet. NC, scramble siRNA; data shown are means ± SD; *n* = 3; mtDNA, mitochondrial DNA; nDNA, nuclear DNA; **p < 0.01, ***p < 0.001 versus the control group.

### 3.5 ECM‐Induced Downregulation of FANCD2 Mediates IFN‐I Expression

Given the crucial role of FANCD2 in maintaining DNA replication fork stability in both the nucleus and mitochondria (Emam et al. 2022; Luzwick et al. 2021), we purified mitochondrial and nuclear fractions from A549 cells and assessed the effects of ECM on FANCD2 expression. Notably, ECM treatment markedly reduced FANCD2 protein levels in both compartments (Figure 5A). Immunostaining further confirmed a pronounced downregulation of FANCD2 in A549 cells following ECM exposure (Figure 5B). Notably, siRNA‐mediated knockdown of *FANCD2* significantly upregulated the expression of *IFNα2* and *IFNβ1* in NSCLC cells (Figure 5C,D). To determine whether ECM‐induced FANCD2 downregulation contributes to cGAS/STING pathway activation, we examined its effect on TBK1 phosphorylation and IFN‐I expression. As anticipated, *FANCD2* knockdown abolished the ability of ECM to induce TBK1 activation and IFN‐I upregulation (Figure 5E,F), indicating that FANCD2 downregulation is required for ECM‐induced cGAS activation and subsequent IFN‐I production. Given that only ECM treatment at 16 μg/mL moderately reduced the transcriptional level of *FANCD2* in A549 cells (Figure 5G), we next examined whether ECM promotes proteasomal or lysosomal degradation of FANCD2 protein. Notably, MG‐132, but not chloroquine, markedly abolished ECM‐induced FANCD2 downregulation (Figure 5H,I), suggesting that ECM primarily facilitates proteasome‐dependent degradation of FANCD2.

**FIGURE 5:**
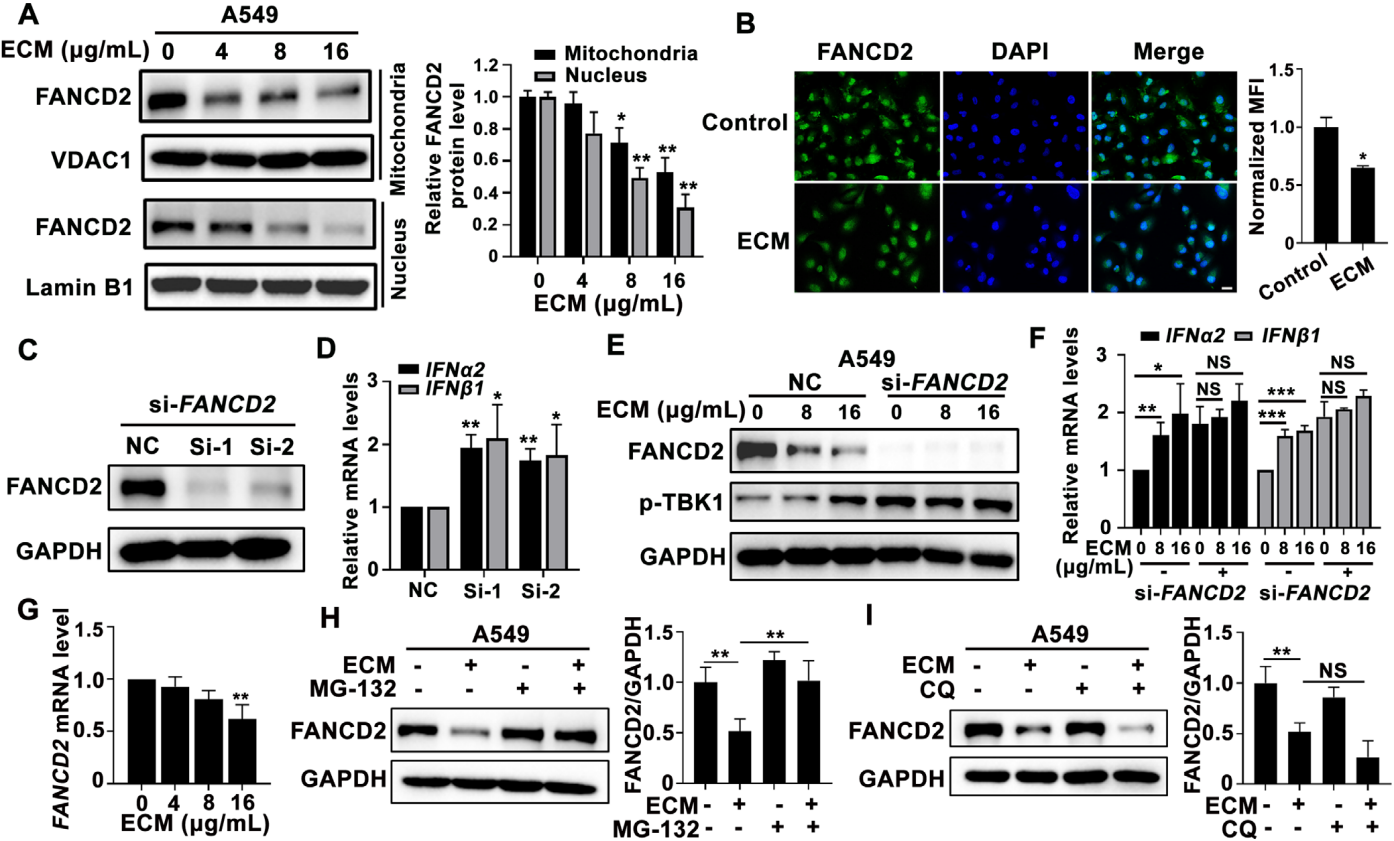
ECM downregulates FANCD2 to increase IFN‐I expression. (A) Mitochondria and nucleus were isolated from A549 cells treated with ECM for 24 h; Western blotting was performed to examine protein levels of mitochondrial and nuclear FANCD2. (B) Immunofluorescence assay for FANCD2 was performed in A549 cells treated with ECM (16 μg/mL) for 24 h; MFI: mean fluorescence intensity. (C, D) After NC or *FANCD2* siRNA transfection for 48 h, Western blotting was performed to examine FANCD2 expression in A549 cells (C); qRT‐PCR was carried out to examine *IFNα2* and *IFNβ1* mRNA levels in A549 cells (D). (E and F) After NC or si‐*FANCD2* (si‐1) transfection for 24 h, A549 cells were treated with ECM for an additional 24 h; the protein levels of FANCD2 and p‐TBK1 were examined by Western blotting (E); *IFNα2* and *IFNβ1* mRNA levels were examined by qRT‐PCR (F). (G) mRNA expression level of FANCD2 was determined in A549 cells after ECM treatment for 24 h. (H, I) A549 cells were treated with ECM (16 μg/mL) for 12 h, followed by the co‐treatment with MG‐132 (10 μM) or CQ (50 μM) for an additional 12 h; FANCD2 protein level was examined by Western blotting. CQ, chloroquine. Scale bars: 20 μm; *n* = 3; NS, not significantly different; *p < 0.05, **p < 0.01, ***p < 0.001 versus the control group.

### 3.6 ECM Synergizes With Anti‐PD‐1 to Enhance Antitumor Efficacy in LLC Syngeneic Model

IFN‐I are known to regulate PD‐L1 expression in tumor cells and PD‐1 transcription in T cells (Taniguchi et al. 2022; Terawaki et al. 2011); therefore, cGAS or STING agonists can substantially improve the efficacy of ICIs. Transcriptomic analysis revealed that ECM treatment significantly upregulated *PD‐1* and other exhaustion markers in CD8^+^ T cells within LLC syngeneic tumor models (Figure S4A), whereas no obvious effects on PD‐L1 upregulation was observed in tumor tissues (Figure S3B). We therefore evaluated the combined anticancer activity of ECM and anti‐PD‐1 in a subcutaneous LLC syngeneic model. As expected, the combination regimen exerted superior antitumor effects compared with either monotherapy, as evidenced by significantly reduced tumor weight and volume (Figure 6A–C). Notably, the combination treatment synergistically enhanced CD8^+^ T cell infiltration into tumors, which was accompanied by elevated expression of the activation marker IFNγ (Figure 6D,E). Furthermore, combined therapy suppressed the accumulation of MDSCs in the TME (Figure S4C). Consistent with these observations, mRNA levels of *IFNγ* and *GZMB* were markedly increased in tumors from the combination group (Figure 6F). While both monotherapy and combination treatment significantly induced *IFN‐I* expression, the combination group exhibited substantially higher *IFN‐I* levels than monotherapy (Figure 6G), suggesting that elevated IFN‐I contributes to CD8^+^ T cell activation in the TME. In line with flow cytometry data, IHC analysis confirmed increased CD8^+^ T cell infiltration and IFNγ expression in tumors from the combination group, along with a pronounced reduction in Ki67‐positive cells (Figure 6H). Importantly, neither ECM nor the combination treatment caused significant changes in body weight or in serum levels of UA, CRE, ALT, or AST (Figure S4B,D). Together, these findings demonstrate that the combination with ECM and anti‐PD‐1 exerts synergistic antitumor activity by promoting IFN‐I‐dependent CD8^+^ T cell infiltration, without inducing obvious systemic toxicity.

**FIGURE 6:**
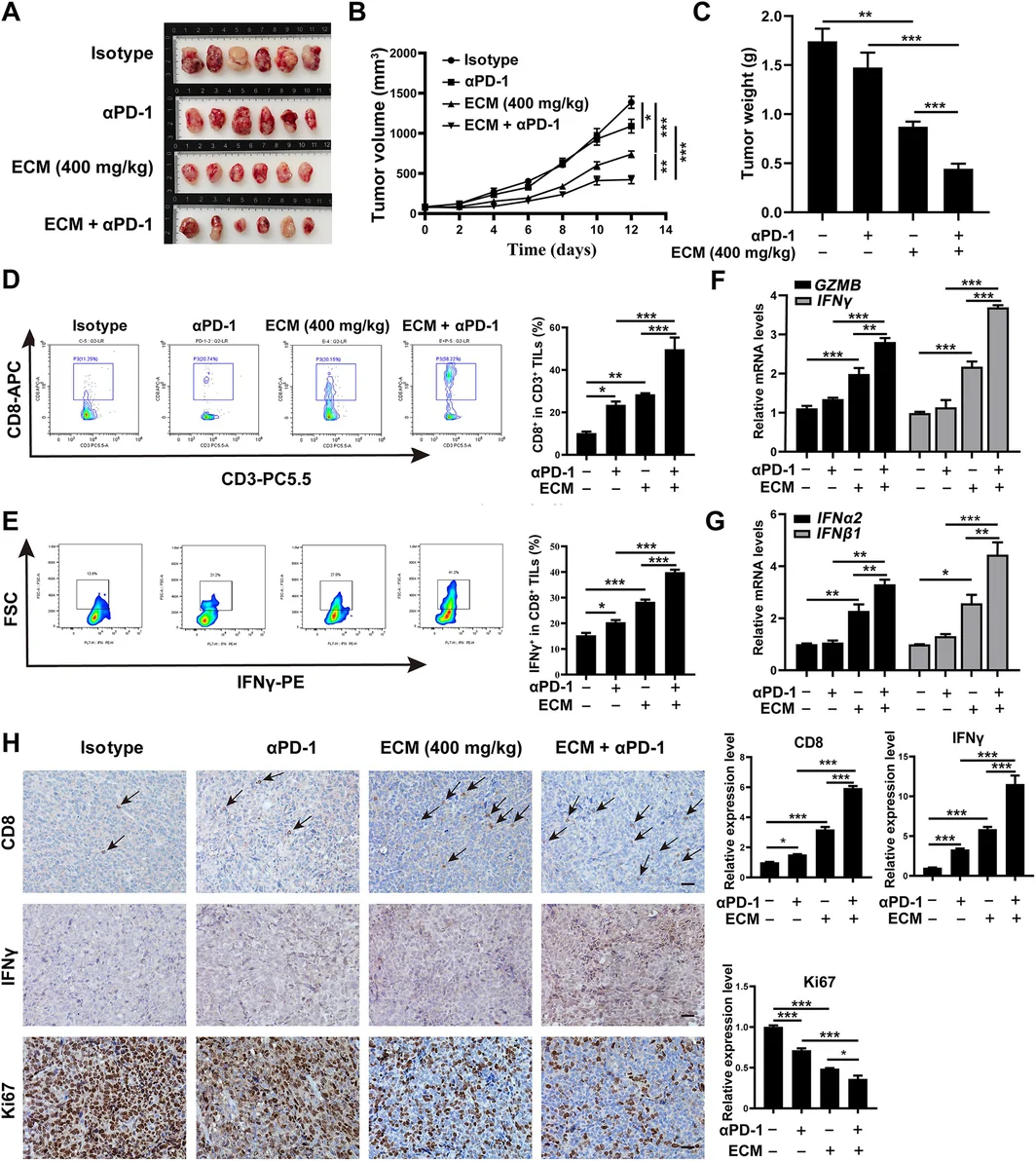
ECM increases tumor infiltrating CD8^+^ T cells and potentiates the therapeutic efficacy of anti‐PD‐1 (αPD‐1). (A) Tumor images were shown at the end of the animal experiment. (B) Tumor volumes were measured every 2 days in indicated groups (*n* = 6). (C) Tumor weights were measured at the end of the experiment (*n* = 6). (D, E) Flow cytometry analysis was performed to show the ratio of tumor infiltrating CD8^+^ T cells among CD3^+^ T cells, and the percentage of IFNγ^+^ in CD8^+^ T cells (*n* = 5). (F, G) qRT‐PCR was carried out to examine the mRNA levels of *GZMB*, *IFNγ*, *IFNα2*, and *IFNβ1* in tumor tissues after drugs treatment (*n* = 5). (H) Representative images from the IHC assay using antibodies against CD8, IFNγ, and Ki67 in tumor tissues of each group; the expression levels of these proteins were quantified by calculating the average optical density using ImageJ software (*n* = 5). Scale bars: 50 μm. Data presented are means ± SEM. *p < 0.05, **p < 0.01, ***p < 0.001.

### 3.7 Identification of the Active Component in ECM

To identify the active constituents of ECM responsible for stimulating the cGAS/STING/IFN‐I pathway, we first performed UPLC‐MS analysis to profile abundant components in ECM. Consistent with previous reports (Chan et al. 2016), comparison of retention times and ion chromatograms between ECM and a standard mixture confirmed the presence of chlorogenic acid, rutin, ICA‐B, ICA‐A, ICA‐C, 3‐OMQ, 6‐OAP, ArC, and ArD as major constituents in ECM (Figure 7A–C).

**FIGURE 7:**
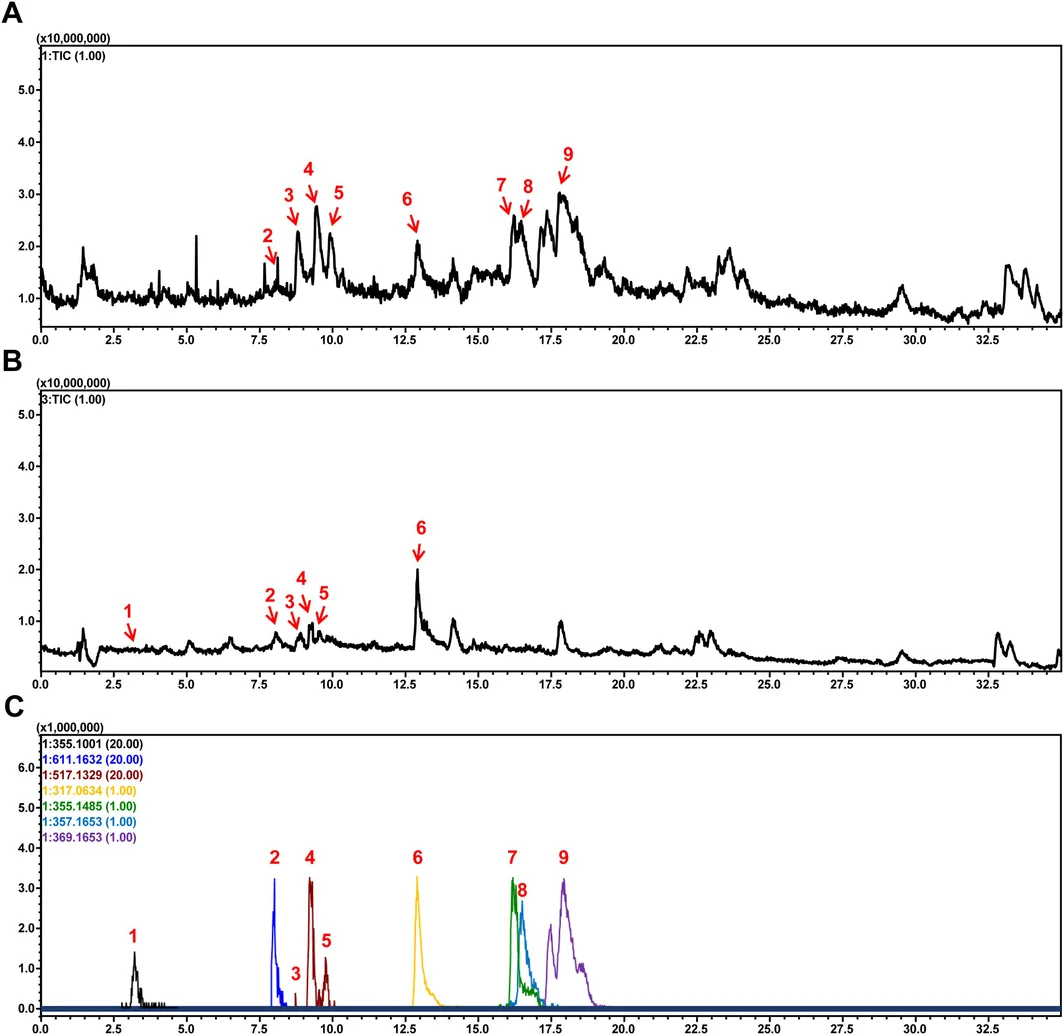
The UPLC‐MS analysis shows these major chemical components in ECM. (A, B) ECM chromatogram in the positive ion mode (A) and negative ion mode (B). (C) The chromatograms extracted from the standard compounds mixture in both the negative and positive ion modes: (1) chlorogenic acid (MW: 354.31, RT: 3.270 min); (2) rutin (MW: 610.52, RT: 8.010 min); (3) ICA‐B (MW: 516.45, RT: 8.805 min); (4) ICA‐A (MW: 516.45, RT: 9.285 min); (5) ICA‐C (MW: 516.45, RT: 9.810 min); (6) 3‐OMQ (MW: 316.26, RT: 12.960 min); (7) ArD (MW: 332.39, RT: 16.155 min); (8) ArC (MW: 334.41, RT: 16.185 min); (9) 6‐OAP (MW: 346.42, RT: 17.895 min).

Based on their relative abundance, five compounds, including ICA‐A, 3‐OMQ, 6‐OAP, ArC, and ArD, were selected to evaluate their potential role in activating the FANCD2‐cGAS/STING signaling axis. Cytotoxicity assays were conducted to determine appropriate concentrations for subsequent experiments. The sesquiterpene lactones 6‐OAP, ArC, and ArD exhibited higher cytotoxicity than 3‐OMQ (Figure 8A,B), while ICA‐A showed no obvious cytotoxicity against LLC cells (Figure S5A). Therefore, sesquiterpene lactones were tested at concentrations below their IC_50_ values, whereas ICA‐A was assessed at relative higher concentrations. For 3‐OMQ, which has been reported to induce G2/M phase arrest without triggering cell apoptosis, concentrations that did not cause obvious cell‐cycle arrest were employed (Figure S5B). Notably, 6‐OAP, ArD, ArC, and 3‐OMQ all markedly enhanced TBK1 and IRF3 phosphorylation (Figure 8C); ICA‐A exhibited no significant effect on the activation of these proteins (Figure S5C). The sesquiterpene lactones and 3‐OMQ enhanced *IFN‐I* expression to varying degrees in NSCLC cells, whereas ArD and 3‐OMQ consistently stimulated the expression of both *IFNβ1* and *IFNα2* in LLC and A549 cells (Figure 8D,E).

**FIGURE 8:**
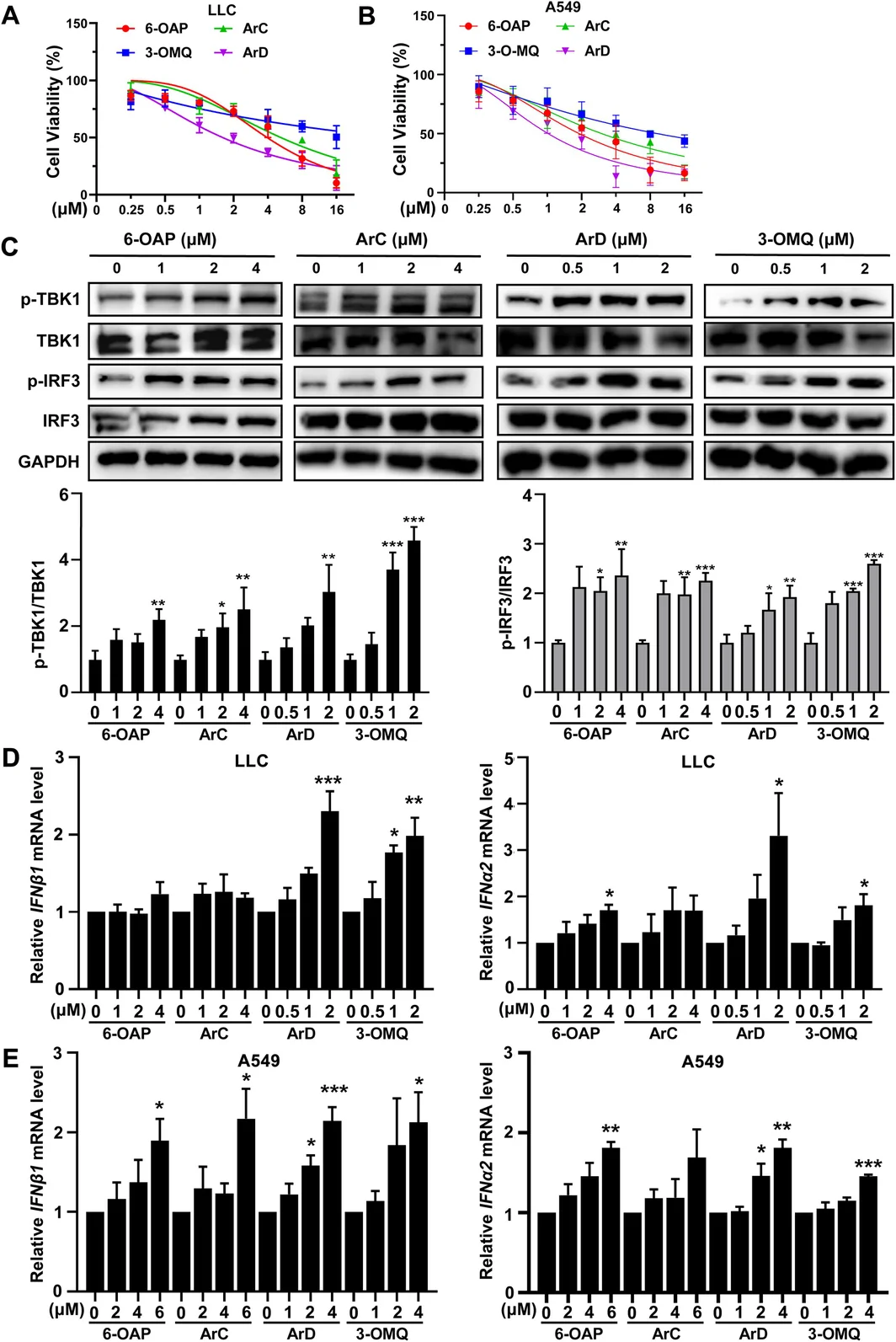
Identification of the active compounds in ECM. (A, B) CCK‐8 assay was performed to examine the inhibitory effects of 6‐OAP, ArC, ArD, and 3‐OMQ on LLC and A549 cell viability after 48 h treatment. (C) Western blotting assay showing protein levels of p‐TBK1 and p‐IRF3 in LLC cells after the treatment of 6‐OAP, ArC, ArD, and 3‐OMQ for 24 h. (D, E) qRT‐PCR assay showing the quantification of the *IFNβ1* and *IFNα2* mRNA levels in LLC (D) and A549 (E) cells after 24 h treatment with 6‐OAP, ArC, ArD, and 3‐OMQ. Data presented are means ± SD. *p < 0.05, **p < 0.01, ***p < 0.001 versus the control group.

As expected, all of these four compounds decreased FANCD2 protein levels in LLC and A549 cells. Notably, ArD decreased FANCD2 protein at a lower concentration (1–2 μM) compared with the other compounds (Figure 9A,B), which was consistent with their relative potency in stimulating *IFN‐I* expression. Based on these observations, the sesquiterpene lactone ArD and the flavonoid 3‐OMQ were selected for further mechanistic investigation into their effects on FANCD2 downregulation. Neither MG‐132 nor chloroquine reversed 3‐OMQ‐induced FANCD2 protein downregulation (Figure 9C,D); however, 3‐OMQ treatment markedly reduced *FANCD2* mRNA levels (Figure 9E), suggesting that 3‐OMQ primarily suppressed *FANCD2* gene expression. In contrast, MG‐132, but not chloroquine, markedly rescued ArD‐induced FANCD2 protein downregulation (Figure 9F,G), demonstrating the capacity of ArD to promote proteasome‐dependent degradation of FANCD2. Taken together, these results indicate that the sesquiterpene lactones and the flavonoid 3‐OMQ may represent the key active components in ECM responsible for activating the FANCD2‐cGAS/STING signaling axis, warranting further investigation into their immunostimulatory and antitumor functions.

**FIGURE 9:**
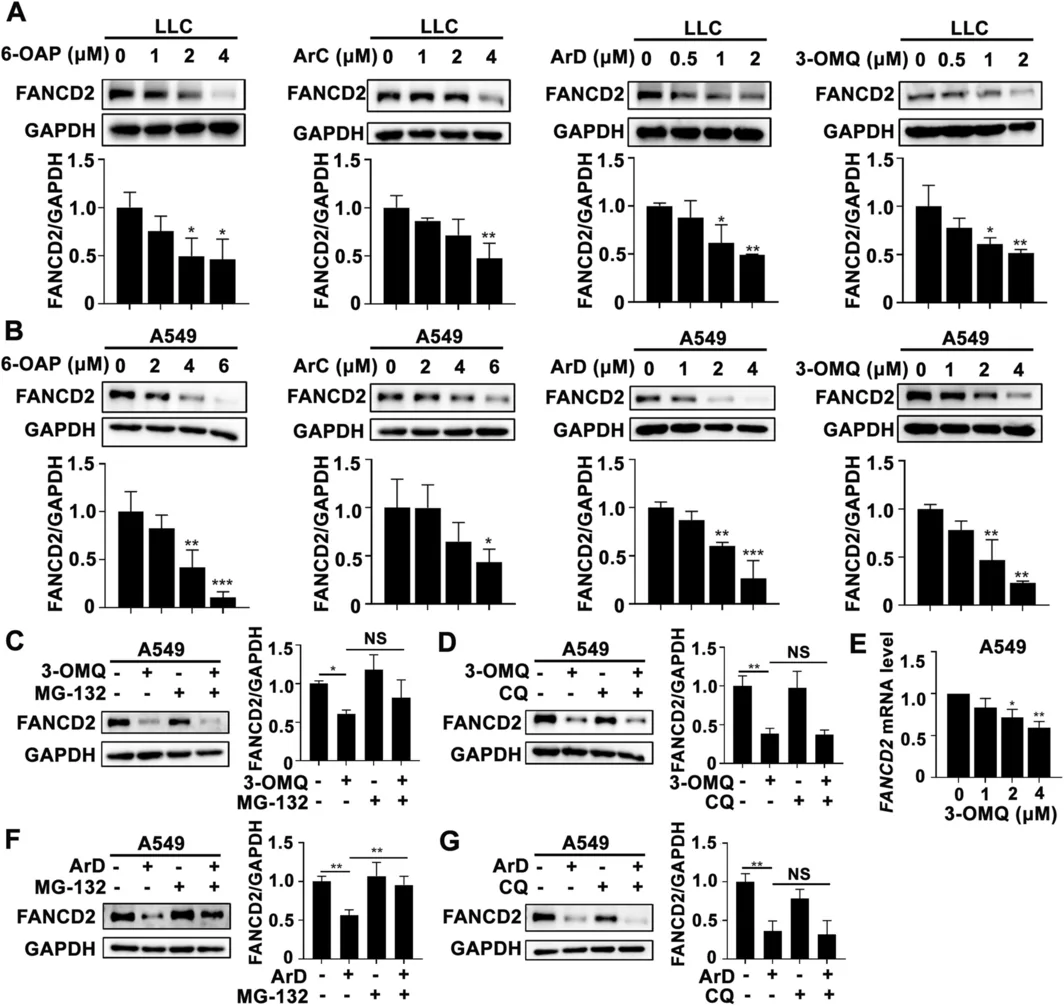
The active compounds in ECM suppress FANCD2 expression. (A, B) After 6‐OAP, ArC, and ArD treatment for 24 h or 3‐OMQ treatment for 48 h, FANCD2 protein levels in LLC and A549 cells were examined by Western blotting. (C, D) A549 cells were treated with 3‐OMQ (4 μM) for 24 h, followed by the co‐treatment with MG‐132 (5 μM) or CQ (50 μM) for an additional 24 h; FANCD2 protein level was examined by Western blotting. (E) mRNA expression level of FANCD2 was determined in A549 cells after 3‐OMQ treatment for 24 h. (F, G) A549 cells were treated with ArD (2 μM) for 12 h, followed by the co‐treatment with MG‐132 (10 μM) or CQ (50 μM) for an additional 12 h; FANCD2 protein level was examined by Western blotting. CQ, chloroquine. Data presented are means ± SD, *n* = 3; NS, not significantly different; *p < 0.05, **p < 0.01, ***p < 0.001.

## 4 Discussion

Patients with NSCLC often exhibit limited response to ICIs, largely attributable to poor infiltration of CD8^+^ T cells within the immunosuppressive TME. Our previous study demonstrated that aqueous extracts of *C. minima* can significantly enhance the efficacy of ICIs (Wang et al. 2024); however, whether *C. minima* promotes CD8^+^ T cell infiltration into the TME and the underlying mechanisms remain unclear. To further delineate the immunomodulatory mechanisms of *C. minima* and explore potential combination strategies, we examined the immune‐activating properties of an ethanol extract of *C. minima* , which exhibits much stronger antitumor effect than the aqueous extract. Our results indicate that ECM stimulates CD8^+^ T cell activation both in vitro and in vivo. RNA‐seq profiling and subsequent in vitro assays revealed that ECM activates innate immunity through the cGAS/STING/IFN‐I signaling pathway, without upregulating PD‐L1. Notably, combined treatment with ECM and anti‐PD‐1 antibody produced synergistic antitumor effects, supporting its potential as a promising combinatorial regimen in NSCLC immunotherapy.

While clinical immunotherapies primarily focus on activating adaptive immunity, the role of innate immunity in antitumor immunotherapy has gained increasing recognition. Multiple therapeutic approaches have been developed to stimulate the cGAS/STING/IFN‐I pathway, which has been shown to promote the infiltration of effector T cells into tumor tissues. Activation of cGAS/STING signaling also contributes to TME reprogramming by enhancing the activity of CD8^+^ T cells, NK cells, and dendritic cells (Lanng et al. 2024). Furthermore, STING‐mediated IFN‐I signaling has been reported to suppress MDSCs differentiation, thereby limiting MDSCs expansion in contexts such as nasopharyngeal carcinoma (Zhang, Ye, et al. 2019). In this study, we provide evidence that ECM markedly activated the cGAS/STING/IFN‐I signaling axis, leading to increased infiltration of CD8^+^ T cells and a reduced proportion of MDSCs in the TME, without significantly altering Treg cell distribution. Notably, although tumor‐infiltrating CD8^+^ T cells exhibited an activated phenotype, marked by elevated expression of CD69, IFNγ, and GZMB, they also displayed upregulated expression of the exhaustive marker PD‐1. Consequently, combining ECM and ICIs resulted in enhanced antitumor efficacy, providing a promising combinatorial strategy for cancer immunotherapy.

Upon activation, TBK1 can phosphorylate both IRF3 and NF‐κB, respectively promoting the production of IFN‐I and inflammatory cytokines (Zhang and Zhang 2025). Consequently, STING agonists that broadly activate this pathway may induce systemic immune activation rather than exerting effects confined to the TME. For example, intravenous administration of the STING agonist diABZI has been shown to stimulate hematopoiesis and alter systemic immune components in peripheral blood and bone marrow (Xu et al. 2023). Moreover, STING agonists carry a substantial risk of triggering acute inflammation through the induction of pro‐inflammatory cytokines such as TNF‐α, IL‐6, and IFNγ (Polidarova et al. 2022), which has limited their clinical translation. In contrast, our study demonstrates that oral administration of ECM did not significantly activate CD8^+^ T in the spleen. Furthermore, ECM treatment neither activated NK‐κB nor upregulated IL‐6 in NSCLC cells, indicating that ECM selectively activates the STING/TBK1/IRF3 innate immune signaling axis while avoiding the systemic adverse effects associated with conventional STING agonists.

Multiple chemotherapeutic agents have been reported to activate the cGAS/STING signaling by inducing mitochondrial or nuclear DNA leakage. Various inhibitors targeting DNA damage repair can substantially promote nuclear DNA release, thereby stimulating cGAS activation (Shen et al. 2019). Similarly, mitochondria‐targeting compounds can trigger mitochondrial DNA leakage and subsequent cGAS activation (Yin et al. 2023). Our previous study has demonstrated that ECM is capable of reducing the protein level of the key DNA repair factor FANCD2, thereby potentiating cisplatin‐induced DNA damage (Fan et al. 2021). Moreover, FANCD2 deficiency leads to loss of replication fork protection in the nucleus, activating the cGAS signaling pathway (Emam et al. 2022). Notably, FANCD2 also plays a role in safeguarding nascent mitochondrial DNA replication forks upon replication stalling (Luzwick et al. 2021). However, whether pharmacological targeting of FANCD2 promotes cGAS/STING/IFN‐I signaling activation and related tumor immunotherapy has not been reported. In the present study, we found that ECM decreases FANCD2 protein levels in both the nucleus and mitochondria while concurrently inducing cytosolic leakage of DNA from both organelles. Knockdown of FANCD2 alone was sufficient to upregulate IFN‐I expression; however, ECM failed to further enhance IFN‐I induction under FANCD2‐deficient conditions, indicating that ECM‐induced FANCD2 downregulation is primarily responsible for activating cGAS/STING signaling.

Four compounds abundant in ECM were shown to downregulate FANCD2 and stimulate TBK1/IRF3 activation. The sesquiterpene lactones, with IC_50_ values ranging from 2 to 8 μM, exhibited relatively higher cytotoxicity than 3‐OMQ; however, 3‐OMQ at concentrations of 1–4 μM can markedly activate the cGAS/STING/IFN‐I signaling pathway and downregulate FANCD2. The sesquiterpene lactone ArD promoted proteasomal‐dependent degradation of FANCD2, whereas 3‐OMQ markedly reduced its gene expression, which is consistent with the dual mechanisms underlying ECM‐mediated downregulation of FANCD2 at both protein and mRNA levels.

Recent pan‐cancer analyses have revealed that FANCD2 expression is significantly upregulated in multiple cancer types (Xie et al. 2024). A comprehensive study across TCGA tumors showed that FANCD2 expression correlates with tumor mutation burden and microsatellite instability (MSI). Moreover, FANCD2 expression was estimated to be negatively correlated with the immune infiltration score in glioblastoma, breast carcinoma, endocervical adenocarcinoma, stomach adenocarcinoma, and lung squamous cell carcinoma (Zhao et al. 2024). Notably, FANCD2 deficiency has been reported to lead to cGAS pathway activation and immune cell migration (Emam et al. 2022), further supporting a close relationship between FANCD2 expression, replication stress, and innate immune activation. In our study, we further demonstrated that the active compounds in ECM, including the sesquiterpene lactones and 3‐OMQ, markedly downregulated FANCD2 and stimulated cGAS/STING signaling activation. Given that FANCD2 may serve as a potential therapeutic target for cancer immunotherapy across multiple tumor types, whether these active compounds can target FANCD2 to enhance innate immune response in other malignancies merits further investigation.

In the present study, oral administration of ECM preferentially promoted effector T cell infiltration into the TME without eliciting obvious side effects, suggesting favorable pharmacokinetic and safety profiles of its active constituents. Among the components highly abundant in ECM, 3‐OMQ and several sesquiterpene lactones were found to downregulate FANCD2 and activate TBK1 and IRF3 in NSCLC cells, indicating their potential role in stimulating the FANCD2‐cGAS/STING signaling axis. Thus, further investigation into the pharmacokinetic properties and in vivo immunomodulatory activities of these active components is warranted.

## 5 Conclusion

ECM and its active components, including 3‐OMQ and the sesquiterpene lactones, were shown to potently activate the FANCD2‐cGAS/STING signaling axis, leading to IFN‐I production in NSCLC cells. Consequently, ECM induced CD8^+^ T cell activation both in co‐culture systems and in the LLC subcutaneous syngeneic model. Moreover, the combination of ECM with anti‐PD‐1 therapy synergistically enhanced the activation of tumor‐infiltrating CD8^+^ T cells, resulting in significant suppression of NSCLC tumor growth.

## Author Contributions

**Han‐Chen Wang:** methodology, software, data curation, investigation, validation, formal analysis, visualization, writing – original draft, conceptualization. **Chu‐Ying Chen:** methodology, data curation, investigation, validation, formal analysis, resources. **Jiang‐Tao Xiao:** data curation, validation, software. **Rou‐Qiao Zheng:** data curation, validation, formal analysis. **Wen‐Da He:** methodology, validation, data curation. **Zi‐Wei Xu:** data curation, visualization. **Su‐Fen Wei:** methodology, data curation. **Tao Su:** data curation, funding acquisition. **Guang‐Biao Zhou:** project administration, funding acquisition, writing – review and editing, resources, conceptualization. **Yong‐Qiang Liu:** conceptualization, data curation, supervision, funding acquisition, project administration, resources, writing – review and editing, writing – original draft.

## Funding

This work was supported by the National Key Research and Development Program of China (2023YFC3503205), National Natural Science Foundation of China (82274158), Guangdong Provincial Key Laboratory of Chinese Medicine for Prevention and Treatment of Refractory Chronic Diseases (2023KT15524), Guangdong Province Modern Agricultural Industrial Technology System Innovation Team Construction Project (2024CXTD24), and the First‐Class Discipline Capacity Enhancement Program of Guangzhou University of Chinese Medicine (GZY2025GB0734).

## Conflicts of Interest

The authors declare no conflicts of interest.

## Supporting information


**Figure S1:** ECM suppresses tumor‐infiltration of MDSCs without affecting Tregs cells in tumor tissues and CD8^+^ T cells in the spleen. (A) Tumor images of LLC cell xenografts. (B) Body weight of the mice was recorded and shown. (C) The flow cytometry gating strategies, including singlet discrimination (SSC‐A vs. SSC‐H), dead cells exclusion using propidium iodide staining, CD45 gating for total leukocyte, CD3 gating for total T cells, and CD11b gating for myeloid‐derived cells. (D) Flow cytometry analysis was performed after staining spleen cells from tumor bearing mice with CD69 and CD8 antibodies (*n* = 5). (E) The total cells isolated from tumor tissues were stained with FOXP3 and CD25 antibodies to perform flow cytometry analysis (*n* = 5). (F) The cells isolated from tumor tissues were stained with CD11b and Gr‐1 to perform flow cytometry analysis (*n* = 5). (G) PCA score plot was shown based on the RNA‐seq data from tumor tissues. Data presented above are shown as means ± SEM, NS: not significantly different; ***p < 0.001 versus control group.
**Figure S2:** ECM enhances the cytotoxicity of activated T cells against NSCLC cell. (A) H1975 cells were pretreated with ECM for 24 h, the viable cells were visualized by crystal violet staining after co‐culture with activated PBMCs for another 24 h, the absorbance of dissolved crystal violet staining of the cells was measured to calculate the percentage of viable cells. (B) Flow cytometry analysis was performed to assess the purity of CD8^+^ T cells isolated from mouse spleen. Data shown are means ± standard deviation (SD). *n* = 3, **p < 0.01, ***p < 0.001 versus control.
**Figure S3:** ECM does not affect PD‐L1 and IL‐6 expression in NSCLC cells. (A) Western blotting analysis was performed to detect PD‐L1 protein levels in H1975, A549, and LLC cells treated with ECM for 24 h. (B) Representative images from the IHC assay using antibodies against PD‐L1 in tumor tissues of vehicle and ECM treatment groups; (C) qRT‐PCR was performed to examine the expression levels of IL6 in LLC cells treated with ECM for 24 h. Scale bars: 25 μm, *n* = 3, NS: not significantly different.
**Figure S4:** The combination with ECM and anti‐PD‐1 suppresses MDSCs tumor infiltration and shows no obvious in vivo toxicity. (A) Heatmap analysis was performed to show gene expression difference of T cell exhaustion markers between control and ECM‐treated tumor tissues. (B) Body weight of mice was measured and presented (*n* = 6). (C) Flow cytometry analysis was performed after staining with CD11b and Gr‐1 antibodies, the percentage of MDSC cells was determined in each group (*n* = 5). (D) At the end of the animal studies, the levels of uric acid (UA), creatinine (CRE), aspartate aminotransferase (AST), and alanine aminotransferase (ALT) in the blood serum of mice in each group were measured (*n* = 6). Data presented above are shown as means ± SEM, NS: not significantly different; **p < 0.01, ***p < 0.001.
**Figure S5:** ICA‐A dose not stimulate TBK1/IRF3 activation and 3‐OMQ induces G2/M phase arrest in LLC cells. (A) CCK‐8 assay was performed to examine the inhibitory effects of ICA‐A on LLC cell viability after 48 h treatment. (B) Flow cytometry analysis was performed to determine cell cycle distribution of LLC cells treated with ECM for 24 h. (C) Western blotting assay showing protein levels of p‐TBK1 and p‐IRF3 in LLC cells after the treatment of ICA‐A for 24 h. Data shown are means ± SD, **p < 0.01 versus control group.

**Table S1:** List of PCR primers used in this study.

## Data Availability

The data that support the findings of this study are available from the corresponding author upon reasonable request.
