# Supplementary material for: Identification of Active Compounds of Centipeda minima for Potentiating Tumor Immunotherapy in Non‐Small Cell Lung Cancer via FANCD2‐cGAS/STING/IFN‐I Signaling Axis
Source: Phytother Res. 2026 Sep 24;40(11):6791–808. doi: 10.1002/ptr.70460 (PMC13753697; doi:10.1002/ptr.70460)
Supplement: Supplementary file 1 [file PTR-40-6791-s001.docx]

**Supplementary information for this article:**

**Identification of Active Compounds of *Centipeda minima* for Potentiating Tumor Immunotherapy in Non-Small Cell Lung Cancer via FANCD2-cGAS/STING/IFN-I Signaling Axis**

**Figure S1.** ECM suppresses tumor-infiltration of MDSCs without affecting Tregs cells in tumor tissues and CD8^+^ T cells in the spleen. (A) Tumor images of LLC cell xenografts. (B) Body weight of the mice was recorded and shown. (C) The flow cytometry gating strategies, including singlet discrimination (SSC-A vs. SSC-H), dead cells exclusion using propidium iodide staining, CD45 gating for total leukocyte, CD3 gating for total T cells, and CD11b gating for myeloid-derived cells. (D) Flow cytometry analysis was performed after staining spleen cells from tumor bearing mice with CD69 and CD8 antibodies (n = 5). (E) The total cells isolated from tumor tissues were stained with FOXP3 and CD25 antibodies to perform flow cytometry analysis (n = 5). (F) The cells isolated from tumor tissues were stained with CD11b and Gr-1 to perform flow cytometry analysis (n = 5). (G) PCA score plot was shown based on the RNA-seq data from tumor tissues. Data presented above is shown as means ± SEM, NS: not significantly different; ****P* ＜ 0.001 versus control group.

**Figure S2.** ECM enhances the cytotoxicity of activated T cells against NSCLC cell. (A) H1975 cells were pretreated with ECM for 24 h, the viable cells were visualized by crystal violet staining after co-culture with activated PBMCs for another 24 h, the absorbance of dissolved crystal violet staining of the cells was measured to calculate the percentage of viable cells. (B) Flow cytometry analysis was performed to assess the purity of CD8^+^ T cells isolated from mouse spleen. Data shown is means ± standard deviation (SD). n = 3, ***P* ＜ 0.01, ****P* ＜ 0.001 versus control.

**Figure S3.** ECM does not affect PD-L1 and IL-6 expression in NSCLC cells. (A) Western blotting analysis was performed to detect PD-L1 protein levels in H1975, A549, and LLC cells treated with ECM for 24 h. (B) Representative images from the IHC assay using antibodies against PD-L1 in tumor tissues of vehicle and ECM treatment groups; (C) qRT-PCR was performed to examine the expression levels of IL6 in LLC cells treated with ECM for 24 h. Scale bars: 25 µm, n = 3, NS: not significantly different.

**Figure S4.** The combination with ECM and anti-PD-1 suppresses MDSCs tumor infiltration and shows no obvious in vivo toxicity. (A) Heatmap analysis was performed to show gene expression difference of T cell exhaustion markers between control and ECM-treated tumor tissues. (B) Body weight of mice was measured and presented (n = 6). (C) Flow cytometry analysis was performed after staining with CD11b and Gr-1 antibodies, the percentage of MDSC cells was determined in each group (n = 5). (D) At the end of the animal studies, the levels of uric acid (UA), creatinine (CRE), aspartate aminotransferase (AST), and alanine aminotransferase (ALT) in the blood serum of mice in each group were measured (n = 6). Data presented above is shown as means ± SEM, NS: not significantly different; ***P* ＜ 0.01, ****P* ＜ 0.001.

**Figure S5.** ICA-A dose not stimulate TBK1/IRF3 activation and 3-OMQ induces G2/M phase arrest in LLC cells. (A) CCK-8 assay was performed to examine the inhibitory effects of ICA-A on LLC cell viability after 48 h treatment. (B) Flow cytometry analysis was performed to determine cell cycle distribution of LLC cells treated with ECM for 24 h. (C) Western blotting assay showing protein levels of p-TBK1 and p-IRF3 in LLC cells after the treatment of ICA-A for 24 h. Data shown is means ± SD, ***P* ＜ 0.01 versus control group.
