# Supplementary material for: Identification of Active Compounds of Centipeda minima for Potentiating Tumor Immunotherapy in Non‐Small Cell Lung Cancer via FANCD2‐cGAS/STING/IFN‐I Signaling Axis
Source: Phytother Res. 2026 Sep 24;40(11):6791–808. doi: 10.1002/ptr.70460 (PMC13753697; doi:10.1002/ptr.70460)
Supplement: Supplementary file 2 [file PTR-40-6791-s002.docx]

**Table S1:** List of PCR primers and siRNA sequences used in this study**.**

| **Gene** | **Forward (5’-3’)** | **Reverse (5’-3’)** |
| --- | --- | --- |
| *β-Actin* (mouse) | GTGGGCCGCTCTAGGCACCAA | CTCTTTGATGTCACGCACGATTTC |
| *CD8α* (mouse) | TTCTGTCGTGCCAGTCCTTC | TGGGACATTTGCAAACACGC |
| *CD69* (mouse) | AGGCTTGTACGAGAAGTTGGA | AGTTCACCAGAATATCGCTTCAG |
| *IFNα2* (mouse) | ATGAGGAGGCTCCCCTTTCTC | AGTAAGATCTCGCAGCACAGG |
| *IFNβ1* (mouse) | CTTCTCCGTCATCTCCATAGGG | CACAGCCCTCTCCATCAACT |
| *GZMB* (mouse) | GAAGATCCTCCTGCTACTGCTG | GCACAAAGTCCTCTCGAATAAGG |
| *IFNγ* (mouse) | CTTGAAAGACAATCAGGCCATC | CAGCAGCGACTCCTTTTCC |
| *β-Actin* (human) | ATCTGGCACCACACCTTCTAC | CAGCCAGGTCCAGACGCAGG |
| *IFNα2* (human) | CTTGTGCCTGGGAGGTTGTC | AAAAGGTGAGCTGGCATACGA |
| *IFNβ1* (human) | CAGGAGAGCAATTTGGAGGA | CTTTCGAAGCCTTTGCTCTG |
| *COX1* (mouse) | GCCCCAGATATAGCATTCCC | GTTCATCCTGTTCCTGCTCC |
| *D-loop* (mouse) | TCCTCCGTGAAACCAACAA | AGCGAGAAGAGGGGCATT |
| *ND1*  (mouse) | CTAGCAGAAACAAACCGGGC | CCGGCTGCGTATTCTACGTT |
| *18s* (mouse) | TAGAGGGACAAGTGGCGTTC | CGCTGAGCCAGTCAGTGT |
| *STING* siRNA-1 (mouse) | GUACCUAGCACUUCACCUAGC |  |
| *STING* siRNA-2(mouse) | CGAAAUAACUGCCGCCUCA |  |
| *FANCD2* siRNA-1 (human) | UACCUCAAGUGUAUCCAUG |  |
| *FANCD2* siRNA-2 (human) | GGAGAUUGAUGGUCUACUA |  |
